# Assessment of Diabetes Mellitus Retrieving Potential of Low Caloric Jamun ( *Syzygium cumini* ) Drink Through Animal Modeling

**DOI:** 10.1002/fsn3.70251

**Published:** 2025-05-05

**Authors:** Huma Saeed, M. Asim Shabbir, Ahmad Rafi, Ahmad Din, Moazzam Rafiq Khan, Poonam Gill, Muhammad Afzaal, Faiyaz Ahmed, Md Faruque Ahmad, Noor Akram, Gebremichael Gebremedhin Hailu

**Affiliations:** ^1^ National Institute of Food Science and Technology University of Agriculture Faisalabad Faisalabad Pakistan; ^2^ Department of Food Science Government College University Faisalabad Faisalabad Pakistan; ^3^ Department of Basic Health Sciences, College of Applied Medical Sciences Qassim University Buraydah Saudi Arabia; ^4^ Department of Clinical Nutrition, College of Nursing and Health Sciences Jazan University Jazan Saudi Arabia; ^5^ Food Safety & Biotechnology Lab, Department of Food Science Government College University Faisalabad Faisalabad Pakistan; ^6^ Food Technology and Process Engineering Oda Bultum University Chiro Ethiopia

**Keywords:** blood glucose, body weight, diabetes, histopathology, jamun drink, low caloric

## Abstract

Diabetes mellitus, a metabolic disorder characterized by persistent hyperglycemia, impacts millions worldwide. Although *Syzygium cumini* L. (Jamun) has been traditionally utilized for its antidiabetic properties, its efficacy remains underexplored. This study aimed to assess the hypoglycemic potential of a low‐calorie Jamun drink in streptozotocin (STZ)‐induced diabetic Sprague Dawley rats. Twenty rats were allocated into four groups: G_0_ (normal feed; negative control), G_1_ (normal feed + STZ at 60 mg/kg BW, administered intraperitoneally; positive control), G_2_ (normal feed + STZ + Jamun sugar drink, 2 mL/day), and G_3_ (normal feed + STZ + Jamun stevia drink, 2 mL/day). Body weight and blood glucose levels were monitored every 15 days over a 45‐day period, while renal and pancreatic tissues were microscopically examined for potential adverse effects. Significant weight gain was observed in G_0_ (120.2 ± 25.37 g to 135.0 ± 17.16 g) and G_3_ (120.8 ± 18.15 g to 132.6 ± 26.28 g). Notably, blood glucose levels in G_2_ and G_3_ decreased significantly, with final Mean ± SD values of 211.8 ± 8.11 mg/dL and 183.6 ± 8.53 mg/dL, respectively. Histological analysis showed no adverse effects on renal or pancreatic tissues in the treatment groups, while the diseased group exhibited marked irregularities and necrosis in both organs. In conclusion, the low‐calorie Jamun drink demonstrated significant potential in reducing blood glucose levels without causing adverse organ effects, suggesting its promise as a functional dietary intervention for diabetes management.

## Graphical abstract

Effect of low caloric Jamun drink on Streptozotocin‐induced diabetes in Sprague Dawley rats.

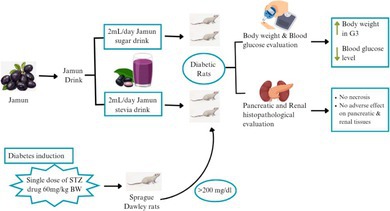

## 1 Introduction

Diabetes mellitus (DM), a common illness that affects 10% of people worldwide, is a major public health issue and is commonly known as the “third killer” (Rather et al. 2019). It is linked to decreased peripheral sensitivity and insulin secretion as well as β‐cell malfunctioning (Ohiagu et al. 2021). DM is a leading cause of heart‐related mortality, blindness, kidney failure, anxiety or depression, and death (Azeem et al. 2022). The International Federation of Diabetes (IDF) estimates that 26.7% of Pakistan's adult population would have diabetes in 2022, with a total of around 33,000,000 cases. An increase in unhealthy lifestyles, population aging, adult, and pediatric obesity, as well as urbanization and economic growth can result in quickly raising the prevalence of diabetes (Silva et al. 2020). Consuming natural antioxidants derived from plants is linked to a lower risk of developing DM, according to epidemiological research (Sun et al. 2021).

Fruits are an essential part of a healthy diet since they are high in fiber, vitamins, and minerals. Their phytochemicals, antioxidants, and phenolic substances contribute to several health‐curative effects (Akonor 2020). Jamun ( *Syzygium cumini* ) native to the subcontinent, is a member of the Myrtaceae family. The majority of jamun cultivation occurs in Asian nations like Pakistan, India, Bangladesh, and Sri Lanka (Joshi et al. 2019; Rizvi et al. 2022). Jamun's abundant supply of bioactive compounds, such as flavonoids, lipids, alkaloids, terpenoids, tannins, and phenols, incorporates a huge potential supply of nutritional and medicinal products with positive effects on health (Rehaman 2021). The presence of these substances exhibits pharmacological activities in human health and metabolism, including antioxidant, antibacterial, antidiabetic, anti‐inflammatory, anti‐allergic, and hepatic protective properties (Tak et al. 2022). The presence of anthocyanins and ellagic acid in jamun has been shown to maintain the ability to prevent starch from being converted to sugar if more glucose is produced, thus exhibiting an antidiabetic property. The most common use of jamun is as an adjuvant treatment for Type 2 diabetes (T2D) (Singh et al. 2019).

Stevia is a natural sweetener, free of calories obtained from leaves of *Stevia rebaudiana Bertoni*. It can be used as a sugar substitute and is safe for those who want to regulate their blood sugar (Basit et al. 2019). It contains chemical compounds known as “steviol glycosides” that have been linked to the release of glucagon‐like peptide‐1 (GLP‐1), a hormone involved in glucose control and insulin secretion, according to some research. Improved blood sugar regulation may be facilitated by elevated GLP‐1 levels (Noya‐Leal et al. 2023).

Jamun pulp's potential health advantages have drawn attention to its value addition in addition to its distinct flavor. That is why a variety of goods with improved nutritional value and a broad variety of flavors are being created, including jam, leather, ready‐to‐serve beverages, and squash (Ayenampudi et al. 2022). Additionally, preserving perishable fruit for an extended period can be achieved by using ready‐to‐serve fruit drinks (Pinto et al. 2022). *Syzygium cumini* (Pomposia), an aromatic plant from the Myrtaceae family, is widely recognized for its traditional and pharmacological benefits. Gas chromatography–mass spectrometry (GC/MS) analysis of the leaf essential oil identified 53 compounds, accounting for approximately 91.22% of the total oil composition. Based on these findings, *S. cumini* oil holds potential as a dietary supplement, particularly for enhancing memory performance in the elderly and supporting blood glucose regulation in diabetic individuals (El‐Nashar et al. 2021). *Syzygium cumini* L., an evergreen tree from the Myrtaceae family, is traditionally utilized for managing diabetes, inflammation, and fever. This study compared the chemical compositions of essential oils (EOs) extracted from various parts of *S. cumini* , including leaves (Scl), fruits (Scf), seeds (Scs), and bark (Scb), using GC/MS analysis. The oils derived from Scl and Scs exhibited notable anti‐collagenase, anti‐elastase, and anti‐hyaluronidase activities. These findings suggest that *S. cumini* oils hold promise as potential ingredients in cosmetic formulations to mitigate signs of skin aging (Ashmawy et al. 2023). A study was conducted to develop electrospun nanofibers loaded with plant extracts as effective wound dressing scaffolds for topical wound healing. Nanofibers incorporating *Syzygium cumini* extract at concentrations of 0.5% and 1% w/v demonstrated enhanced antibacterial activity against tested Gram‐positive bacteria. Additionally, in vivo wound healing was assessed in Wistar rats over a 14‐day period. The results revealed that nanofiber mats containing *S. cumini* leaf extract (SCLE) and collagen significantly accelerated wound healing compared to the untreated control group. These findings underscore the potential of the fabricated nanofibers as promising candidates for promoting wound healing and managing acute topical wounds (Abdelazim et al. 2024).

The goal of the present research is to minimize postharvest losses by converting the fruit into a valuable nutritious product that will be a good source for the diabetic community of Pakistan. The current experiment is intended to evaluate the antidiabetic potential of low calorie jamun drink, as well as its impact on body weight, blood glucose level, and pancreas and kidney histology in an animal model. So, the study will provide good knowledge about the use of low calorie jamun drink in clinical research.

## 2 Materials and Methods

### 2.1 Procurement of Raw Material

Jamun ( *Syzygium cumini* ) was procured from the Ayub Agriculture Research Institute of Faisalabad, Pakistan, while CMC, xanthan gum, sodium citrate, sodium benzoate, and citric acid were obtained from New Chemical Store, Lahore. The pulp of jamun fruit was extracted at the Beverages Laboratory, University of Agriculture, Faisalabad, Pakistan.

### 2.2 Preparation of Jamun Drink

All chemicals were measured accurately. Initially, raw jamun pulp (80 mL/1 L) was blended to obtain desirable fine pulp and homogenized with water (920 mL/1 L). Carboxy methyl cellulose (CMC) (0.5 g/1 L) and xanthan gum (1 g/1 L) were blended in lukewarm water to minimize sedimentation and phase separation in the final product. Fresh blended pulp, CMC, and gum were first pasteurized at 85°C–90°C for 5 min and then mixed with stevia (0.5 g)/ sugar (0.5 g), sodium citrate (0.5 g), citric acid (1.1 g) and sodium benzoate (0.7 g) at low flame. Then, flavor (1 mL) and color (1.1 g) were added to 1 L of distilled water. Filtration was carried out using muslin cloth, and it was filled into pre‐sterilized glass bottles. Finally, the bottles were stored at room temperature.

### 2.3 Experimental Design

The efficacy trial was conducted to evaluate the effect of sugar and stevia‐based low‐calorie jamun drink on diabetic biomarkers and histology. For this purpose, 20 female Sprague Dawley rats (120–170 g) were housed in an animal room and kept for 45 days at the National Institute of Food Science and Technology, University of Agriculture, Faisalabad, Pakistan. All rats were provided with standard conditions including ventilation facility, a 12/12‐h period of light‐dark cycle, and a temperature of the room about (22°C ± 2°C). Prior to the research experiment, ethical approval was obtained from the Office of Research, Innovation and Commercialization. The ethical approval was obtained from the Directorate of Research and Innovation, University of Agriculture Faisalabad (Reference number 1317/ORIC dated 10‐01‐2024). The rats were given a normal diet for a week to acclimatize them; moreover, an environmental condition was maintained with 23°C ± 2°C and 55% ± 5% relative humidity. The rats were categorized into four groups, each group having five rats, and the grouping of rats is mentioned in Table 1. The formulated normal diet for rats was comprised of protein (10%), minerals (3%), corn oil (10%), corn starch (66%), cellulose (10%), and vitamin mixture (1%). A single intraperitoneal injection of streptozotocin (STZ) was used to induce diabetes in rats at a dose of 60 mg/kg/BW, and the animals were allowed to drink 5% dextrose solution overnight to overcome the drug‐induced hypoglycemia. After 3 days, blood glucose levels were measured, and the animals with a glucose concentration of more than 200 mg/mL were classified as diabetic.

**TABLE 1:** Diet and treatment plan for animal trial.

Groups	Treatments
G_0_: Negative control (normal rats)	Normal diet
G_1_: Positive control (diabetic rats)	Normal diet + STZ
G_2_: Treatment I (Diabetic rats)	Normal feed + STZ + 2 mL/day jamun nectar with sugar
G_3_: Treatment II (Diabetic rats)	Normal feed + STZ + 2 mL/day jamun nectar with stevia

### 2.4 Biochemical Analysis

Electronic glucometer was used to measure blood sugar level at 15, 30, and 45th day. A single droplet of blood from the tail vein placed on a strip gadget provides a readout in 2–5 s. During treatment, rats weight was measured using a weighing machine.

### 2.5 Histopathology Evaluation

Rats were dissected for this purpose, and the liver and kidney were sliced into smaller fragments and kept in formalin for 3 days. The tissues were dried after being cut to a width of 0.3–0.5 cm 3.13.3. The dried samples were infiltrated with histology wax molds and left in a chloroform solution for the entire night. Using a razor blade, extra paraffin was removed after it solidified. The slides were soaked in xylene for three to 4 min, and then they were dipped in Erlich's Hematoxylin dye for 30 min to provide the tissue sections contrast and make the tissues visible for microscopic inspection. To prepare the slides for the examination, the coverslip was then placed over the tissue.

### 2.6 Statistical Analysis

The statistical analysis for parameters measured and the blood biochemistry before and after treatment was analyzed using a TWO‐WAY ANOVA test followed by a Factorial Design with a significance value of *p* < 0.005. All the obtained results are expressed as mean ± SD.

## 3 Results

### 3.1 Body Weight of Normal and Diabetic Rats Treated With Jamun Sugar Drink and Jamun Stevia Drink

The statistical analysis showed that there was a significant effect of treatments and days on the body weight (*p* < 0.05). Means of body weight of rats are drawn in Table 2. The body weight of G_0_, which is the control group, noticed at day zero was 120.2 ± 25.37 g and at 45th day 135.0 ± 17.16 g. The body weight of G_1_ (diabetic group) and G_2_ (diabetic group treated with jamun sugar drink) rats was reduced from 150.8 ± 12.67 g to 119.2 ± 21.89 g and from 136.0 ± 16.68 g to 126.8 ± 32.51 g, respectively, from 0 to 45th day. While the body weight of G_3_ (diabetic group treated with jamun stevia drink) was increased from 120.8 ± 18.15 g to 132.6 ± 26.28 g from 0 to 45th day. According to the results, the mean ± SD value of body weight was increased significantly in G_0_ and G_3_, while the mean ± SD value of body weight in G_1_ and G_2_ was significantly reduced.

**TABLE 2:** Effect of treatments on body weight (grams) of rats.

Groups	Days				
	0	15	30	45	Means
G_0_ = Negative control (normal rats)*	120.2 ± 25.37	124.8 ± 21.78	130.6 ± 15.07	135.0 ± 17.16	127.65 ± 19.84^b^
G_1_ = Positive control (Normal diet + STZ)Positive control (Normal diet + STZ)	150.8 ± 12.67	145.4 ± 13.63	117.6 ± 18.95	119.2 ± 21.89	133.25 ± 16.78^a^
G_2_ = Treatment I (Normal feed + STZ + 2mL/day jamun nectar withsugar)	136.0 ± 16.68	132.0 ± 16.04	123.2 ± 31.12	126.8 ± 32.51	129.50 ± 24.08^ab^
G_3_ = Treatment II (Normal feed + STZ + 2mL/day jamun nectarwith stevia)	120.8 ± 18.15	123.0 ± 17.22	128.6 ± 19.69	132.6 ± 26.28	126.25 ± 20.33^ab^
Means	131.95 ± 18.21^ab^	131.30 ± 17.16^a^	125.0 ± 21.20^b^	128.40 ± 24.46^ab^	

* Values are presented as Mean ± standard deviation; different superscript letters (a, b, ab) within the same column are significantly different at *p* < 0.05.

### 3.2 Blood Glucose Level (BGL) of Normal and Diabetic Rats Treated With Jamun Sugar Drink and Jamun Stevia Drink

The results showed a highly significant effect of treatments and days on blood glucose level (*p* < 0.01). Means of blood glucose level of rats are drawn in Table 3. The glucose level of G_0_ noticed at day zero was 94.2 ± 15.46 mg/dL and at 30th day was 88.2 ± 11.73 mg/dL. The mean blood glucose level of G_2_ and G_3_ rats was reduced from 259.6 ± 9.44 mg/dL to 211.8 ± 8.11 mg/dL and 254.6 ± 9.86 mg/dL to 183.6 ± 8.53 mg/dL, respectively, from 0 to 30th day. While the glucose level of G_1_ was unchanged with a range from 272.4 ± 5.85 mg/dL to 273.4 ± 13.06 mg/dL from 0 to 30th day. According to observation, the mean ± SD of blood glucose level was decreased significantly in the group fed with jamun stevia drink.

**TABLE 3:** Effect of treatments on blood glucose level (mg/dL) of rats after induction of diabetes.

Groups	Days			
	0	15	30	Means
G_0_ = Negative control (normal rats)*	94.2 ± 15.46	100.2 ± 16.31	88.2 ± 11.73	94.20 ± 14.5^d^
G_1_ = Positive control (Normal diet + STZ)Positive control (Normal diet + STZ)	272.4 ± 5.85	279.8 ± 5.89	273.4 ± 13.06	275.20 ± 8.26^a^
G_2_ = Treatment I (Normal feed + STZ + 2mL/day jamun nectar withsugar)	259.6 ± 9.44	241.0 ± 10.36	211.8 ± 8.11	237.46 ± 9.30^b^
G_3_ = Treatment II (Normal feed + STZ + 2mL/day jamun nectarwith stevia)	254.6 ± 9.86	219.4 ± 9.81	183.6 ± 8.53	219.20 ± 9.4^c^
Means	220.20 ± 10.15^a^	210.10 ± 10.59^b^	189.25 ± 10.35^c^	

* Values are presented as Mean ± standard deviation; different superscript letters (a, b, ab) within the same column are significantly different at *p* < 0.05.

### 3.3 Pancreatic and Renal Histopathological Evaluation

**FIGURE 1:**
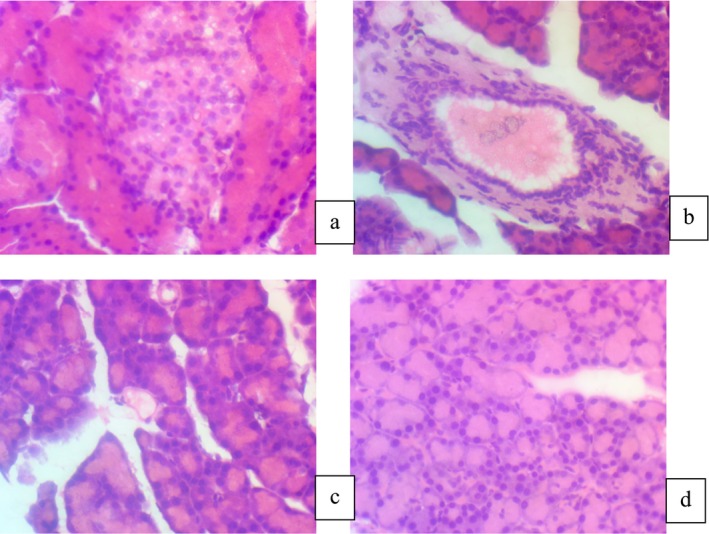
Histopathological indications of pancreatic tissue. (a) (G_0_) Negative Control, (b) (G_1_) Positive Control diabetic rats + Normal diet, (c) (G_2_) Normal feed + STZ‐drug + Jamun sugar drink (2 mL/day), (d) (G_3_) Normal feed + STZ‐drug + Jamun stevia drink (2 mL/day).

Figure 1 shows the islet of Langerhans in the control group (G0) rats that were fed with a normal diet. The histopathology of the pancreas of the control group depicted that the islet shows a high number of beta cells dispersed throughout the islet (Figure 1a).

Whilst in diabetic rats, the disease was induced in rats using streptozotocin (G_1_). The histopathological examination predicts that a fall in the number of beta cells was observed as compared to that in the normal control group rats (Figure 1b). The degeneration of the beta cells was caused by the streptozotocin used to trigger diabetes.

In the third group (G_2_), the streptozotocin‐induced diabetic rats were treated with jamun sugar drink (2 mL/day). The histopathology of kidney tissues indicated the partial repair of islets of Langerhans, as shown in (Figure 1c).

Group four (G_3_) had diabetic rats and were fed 2 mL/day jamun stevia drink. During the microscopic study, treatment with jamun stevia drink significantly increased the reduction of Langerhans islet diameter, as shown in (Figure 1d).

**FIGURE 2:**
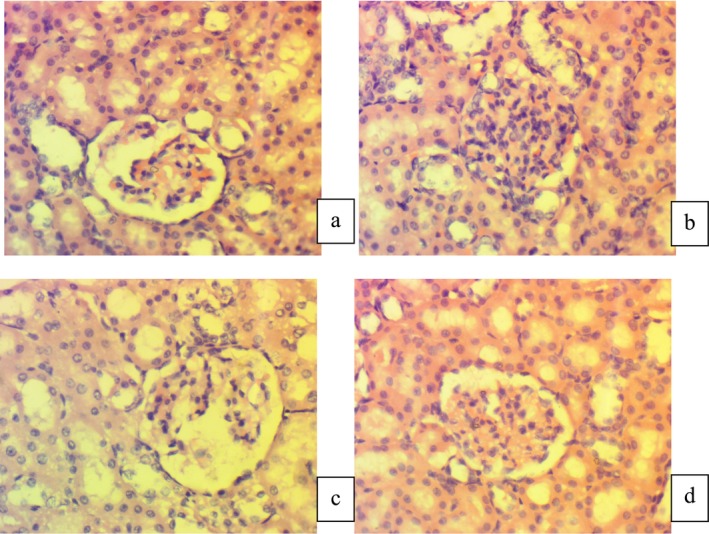
Histopathological indications of Kidney tissue. (a) (G_0_) Negative Control, (b) (G_1_) Positive Control diabetic rats + Normal diet, (c) (G_2_) Normal feed + STZ‐drug + Jamun sugar drink (2 mL/day), (d) (G_3_) Normal feed + STZ‐drug + Jamun stevia drink (2 mL/day).

Figure 2 showed the histopathology of kidney tissues of the control group (G0) depicted the normal appearance of the renal parenchyma. Moreover, the tissues had normal benign‐looking tubules with well‐preserved architecture lined by cuboidal epithelial cells with centrally placed round to oval nuclei (Figure 2a).

Whilst in diabetic rats (G_1_), the kidney tissues showed inflammation and necrotic changes at the cellular level that predicted a mild level of toxicity of streptozotocin on the renal tissues. The overall cellular architecture was also compromised (Figure 2b).

In the third group (G_2_), the streptozotocin‐induced diabetic rats were treated with jamun sugar drink (2 mL/day). The histopathology of kidney tissues indicated that the cells had a normal architecture. The kidney tubules had some necrotic foci at some spots, but the overall structure of the kidney tubules was preserved (Figure 2c).

The group four (G_3_) had diabetic rats and was fed with 2 mL/day jamun stevia drink. According to the microscopic evaluation, there was no definite inflammation and necrotic areas in the kidney tubules and the cellular boundaries were intact (Figure 2cd).

## 4 Discussion

Diabetes is one of the fastest growing diseases worldwide, projected to affect 693 million adults by 2045. Devastating macrovascular complications (cardiovascular disease) and microvascular complications (such as diabetic kidney disease, diabetic retinopathy and neuropathy) lead to increased mortality, blindness, kidney failure, and an overall decreased quality of life in individuals with diabetes (Cole and Florez 2020). With the recent lifestyle changes, diabetes is constantly on the rise. Management of patients with diabetes needs inclusion of various methods with special attention to lifestyle modifications like weight loss, exercise, and diet control (Borse et al. 2021). Many ayurvedic plants have been used for the treatment of diabetes. *Syzygium cumini* has been used in traditional ayurvedic medicine since ancient times to treat hyperglycaemia. Availability of many literatures in Ayurveda and modern medicine suggests antihyperglycemic activity of *Syzygium cumini* (Mulkalwar et al. 2021).

One of the significant observations was the effect of the Jamun drink on body weight. Diabetes often causes weight loss due to the catabolic state induced by insulin deficiency (Dmello and Bhagwat 2024). The untreated diabetic group exhibited a marked decrease in body weight. However, the group treated with the Jamun drink showed a significant increase in body weight compared to the untreated diabetic group. This suggests that the Jamun drink helps counteract weight loss associated with diabetes, possibly by improving metabolic efficiency and nutrient utilization (Baliga et al. 2013). The findings of another study align with present research on the in vivo herb‐drug interaction study using jamun seed powder (JSP) and Metformin, which indicated that the concomitant use of these two drugs has no significant effect on the body weight of treated and control rats (Geetika et al. 2022). This effect could be attributed to the high fiber content and other bioactive compounds in Jamun, which enhance digestive health and nutrient absorption.

Analysis of *Syzygium cumini* leaf essential oil revealed a complex composition, identifying 53 compounds that constituted approximately 91.22% of the total oil. These findings highlight the rich phytochemical profile of *S. cumini* oil, which may contribute to its diverse biological activities. According to El‐Nashar et al. (2021), this oil shows significant potential as a dietary supplement, particularly for improving memory performance in elderly individuals and aiding blood glucose regulation in diabetic patients. The study underscores the therapeutic promise of *S. cumini* oil, likely due to its bioactive components with antioxidant, anti‐inflammatory, and neuroprotective properties. These properties may help mitigate oxidative stress—a key factor in cognitive decline and diabetes progression. However, while the in vitro and preliminary findings are promising, further research is needed to validate these effects through clinical trials. Additionally, understanding the mechanisms of action and identifying the specific compounds responsible for these benefits could pave the way for targeted applications in dietary and medical interventions.

Blood glucose levels are a critical indicator of diabetes management. In the study, the untreated diabetic group had significantly elevated blood glucose levels compared to the control group. The administration of the Jamun drink resulted in a significant reduction in blood glucose levels, bringing them closer to normal ranges. This hypoglycemic effect is likely due to the presence of bioactive compounds such as jamboline and ellagic acid in Jamun, which have been shown to enhance insulin secretion and improve insulin sensitivity (Das et al. 2023). The results align with previous studies that have demonstrated the efficacy of Jamun in lowering blood glucose levels and managing diabetes (Imran et al. 2022). Similarly, a study conducted by Darwish et al. (2020) analyzed the effects of *P. peruviana* fruit juice, pomace, and metformin on blood glucose levels in STZ‐induced diabetes rats. At the end of the experiment, the reduction in blood glucose concentration continued and reached 62.6%, 57.6%, and 54.8% for metformin, *P. peruviana* fruit juice, and pomace, respectively. Likewise, the administration of aqueous seed extract of Jamun at a dose of 1 g/kg body weight in diabetic rats has been reported to produce a hypoglycemic effect in the blood (Amin 2020). Phytochemicals present in jamun seeds have a beneficial effect on hypoglycemic activity by stimulating glucose uptake (GLUT 4) by skeletal muscle cells (Syama et al. 2018). Parveen et al. (2020) demonstrated the antihyperglycemic and antihyperlipidemic effect of jamun seeds in a human study. The supplementation of the diet of patients diagnosed with prediabetes with jamun seed capsulated powder (4.5 g/day) significantly improved glucose levels and total cholesterol and LDL‐cholesterol from 266 to 216 mg/dL and from 189 to 139 mg/dL, respectively.

The histopathological evaluation of pancreatic tissues revealed significant findings, particularly in the size and structure of the islets of Langerhans. In the diabetic control group, the islets appeared markedly reduced in size, with evidence of beta‐cell necrosis and disorganization. Conversely, the groups treated with the Jamun drink exhibited larger and more defined islets of Langerhans, indicating a protective effect on pancreatic tissues. This preservation is likely attributed to the antioxidant properties of Jamun, which help mitigate oxidative stress—one of the primary contributors to beta‐cell damage in diabetes (Amin et al. 2023; Kumar et al. 2023). The increased size of the islets in treated groups may suggest an enhancement in beta‐cell regeneration or a reduction in cell apoptosis due to the antioxidative and anti‐inflammatory effects of bioactive compounds in the Jamun extract. These compounds, such as flavonoids and anthocyanins, are known to scavenge free radicals and reduce oxidative damage. This phenomenon supports the hypothesis that the Jamun drink not only protects existing beta cells but may also contribute to the restoration of pancreatic function, thereby improving endogenous insulin production. While these findings are promising, the mechanisms behind the observed preservation and potential regeneration of beta cells warrant further investigation. Studies involving molecular markers of beta‐cell proliferation and apoptosis would provide deeper insights into the role of Jamun in pancreatic tissue repair and insulin homeostasis. Additionally, future research should explore whether these histological improvements correlate with long‐term glycemic control and overall diabetes management in clinical settings.

Diabetic nephropathy is a severe complication of diabetes, leading to chronic kidney disease. The histopathological analysis of renal tissues in the study indicated a positive effect on glomerular and tubular structure. This nephroprotective effect is likely due to the anti‐inflammatory and antioxidant properties of Jamun, which help reduce oxidative stress and inflammation in renal tissues (Akram et al. 2022). By protecting the kidneys from diabetic damage, Jamun could play a crucial role in managing diabetic nephropathy.

The study also compared the effects of the Jamun drink sweetened with sugar and stevia, highlighting potential differences in their impact on diabetes management. Both formulations significantly reduced blood glucose levels, with the stevia‐sweetened drink demonstrating a slightly greater hypoglycemic effect. This observation may be attributed to stevia's natural low‐calorie properties and its potential role in enhancing insulin sensitivity. In contrast, while the sugar‐sweetened drink effectively reduced blood glucose levels, its caloric content could pose a concern for long‐term use in diabetic patients. These findings emphasize the dual benefits of the Jamun drink, the inherent antidiabetic properties of *Syzygium cumini* and the added advantages of stevia as a natural sweetener. Stevia may be a preferable option for individuals requiring strict calorie control, further reinforcing the drink's potential as a functional dietary intervention for diabetes management. Future studies should explore these formulations long‐term metabolic impacts and their role in comprehensive diabetes care.

## 5 Conclusion

This study demonstrated that the low‐calorie Jamun ( *Syzygium cumini* ) beverage holds significant potential for managing diabetes mellitus, as evidenced by its ability to increase body weight, reduce blood glucose levels, and preserve the histological integrity of pancreatic and kidney tissues in diabetic animal models. The observed therapeutic effects are likely attributed to the rich bioactive compounds present in Jamun, known for their anti‐inflammatory and antioxidant properties. Despite these promising findings, the study is limited to a preclinical animal model, and the results may not fully translate to human populations. Additionally, the molecular mechanisms underlying the hypoglycemic and tissue‐protective effects of the Jamun beverage remain unclear and warrant further investigation. Future research should focus on conducting clinical trials to validate these findings in humans and determine the safety and efficacy of the Jamun beverage as a therapeutic intervention. Moreover, mechanistic studies are needed to elucidate the molecular pathways involved in its antidiabetic effects. Exploring the formulation and optimization of the beverage for broader application in dietary management of diabetes would also be valuable.

## Author Contributions

**Huma Saeed:** writing – original draft (equal). **Ahmad Rafi:** methodology (equal), project administration (equal). **Ahmad Din:** validation (equal), visualization (equal), writing – review and editing (equal). **Moazzam Rafiq Khan:** project administration (equal), validation (equal). **Poonam Gill:** data curation (equal), visualization (equal). **Md Faruque Ahmad:** validation (equal), writing – review and editing (equal). **Noor Akram:** writing – review and editing (equal).

## Conflicts of Interest

The authors declare no conflicts of interest.

## Data Availability

Even though adequate data has been given in the form of tables and figures all authors declare that if more data is required then the data will be provided on a request basis.
